# Hospital Progress and Finance

**Published:** 1924-12

**Authors:** 


### HOSPITAL PROGRESS AND FINANCE.

### COMPLAINTS ABOUT DIETARY.

It is curious to observe how delicate a matter is the dietary of a hospital. Once the patients, all of whom oscillate somewhere between the extremity of long-suffering and cantankerousness, begin to take their food without feeling truly thankful, it is hard to say where the pother will stop. There can hardly be an editor of a professional journal who does not receive at regular intervals letters from nurses, probationers and other members of a hospital's staff, drawing attention to the alleged deficiencies in the dietary of his or her hospital or infirmary. These complaints are often well founded, though equally often the real cause of complaint is not the quantity or quality of the food, but the slovenly manner in which it is cooked or served. To remedy such defects is less easy than appears to the complainant. In the first place discipline, not unnaturally, looks with a cold eye upon anyone within an institution who seeks redress from beyond its walls, for such action implies an independence of spirit, valuable indeed to its possessor when not thoughtlessly employed, that may be inconvenient to authorities not statesmanlike enough to see how, when reasonably respected and shown, it fosters a true, and checks a false, *esprit de corps*.

### THE SENSITIVENESS OF AUTHORITY.

To abuse authority is a temptation that few authorities can resist when tempted; so the complainer cannot be called by his outside confidant to give evidence. The authorities, too, are naturally sensitive, especially about diet, for Bumble's ghost still haunts the imagination of many institutional managers.: if someone calls you Bumble, there is no equally effective reply. The reflection cast upon you sounds so simple, so suffocating, and yet is so elusive that you might as well fire a pistol at a fog. The best means, perhaps, of dealing with complaints concerning a dietary is to obtain a copy, and publish it if the complaint appears, from the table, to be sound. When an admittedly good dietary is set side by side with one apparently inferior one, the comparison will tell its own story.

### WHY NOT A RELATIVE UNIFORMITY ?

But even this simple procedure is beset with snares. For example, a sub-committee of the King Edward VII. Welsh National Memorial Committee, having toured the association's sanatoria, lately prepared a report upon the cost of the foodstuffs used in each. Though the cost is a much less sensitive matter than the quality, quantity, or service of a hospital's diet, fears were immediately shown lest the comparisons which gave point to the report would be resented. In defence it was urged (as if to prove the very point that we are emphasising) that the report was unexceptionable because it did not attempt to touch the question of diet! Nevertheless, what the Medical Director called a relative uniformity in the cost of foodstuffs is desirable in any group of similar and comparable institutions. This, of course, is true We wish to point out also that uniformity of dietary is equally desirable in similar hospitals. This is a matter to which the promising local committees, formed in relation to the Voluntary Hospitals Commission, and indeed the regional committees of the British Hospitals Association, would do well to consider. The high standard of hospital and Poor Law infirmary dietary has been taken for granted far too long.

### THE INVESTIGATION AT LOWESTOFT.

The report on the Lowestoft and North Suffolk Hospital, by Dr. F. N. K. Menzies of the British Red Cross Society, has been made to a special meeting of the governors. The report arose from some criticisms made at a previous public meeting and Dr. Menzies was invoked to advise. With only sixty-seven beds out of eighty-seven occupied by patients, the adviser has found the hospital underbedded; a separate nurses' home needed; a resident medical officer highly desirable; a modern X-ray department desirable; also massage, electricity and laboratory facilities. In other respects the hospital has been found well-built and well-designed. Administratively, Dr. Menzies advocated the appointment of a first-class full-time, well-paid secretary; a contributory scheme or payment for work done on behalf of public authorities, together with economy in administration and maintenance. A good organiser, said Dr. Menzies, should be able to double the income of the hospital. Colonel A. G. Lucas, the president, and Lord Somerleyton moved and seconded a vote of thanks, the latter advocating a separate out-patients' department. Experience shows that a similar policy, adopted elsewhere, and loyally carried out will produce the effects that experienced people claim for it.

### A MODEL HOSPITAL BRIEF.

The hill at Harrow, the conspicuous church of which is reputed to have led Charles II. to explain that the author of the phrase " the visible Church " must have had St. Mary's, Harrow, in his mind, is adorned, with other pleasant buildings, by the cottage hospital. A children's ward is now being added to the growing building by the generosity of Mr. and Mrs. J. N. Stuart. The cost, £4,000, is to be made a self-supporting expense by the raising of a similar sum to endow the new ward. To this end an appeal is being made which is a model of directness, good sense, and good taste. A need, after all, is only the vague, emotional, expression of some fact; and if the facts can be presented in such a way as to convey the sense of the need that they create, then there is no necessity for windy rhetoric or wordy padding. The writer of this appeal, presumably, is the unnamed member of the medical staff together with Mr. Lionel M. Hewlett, the honorary secretary; and there can be no question that the writing is extremely good. Does this collaboration explain it ? And, if so, should it become the rule ? We have no room for it all, but the appeal should be studied by all hospital administrators in search of a model. The new wing will contain twelve beds and cots, a sun-trap verandah overlooking the slope whereon the hospital stands, the usual offices, and certain desirable rooms for the nursing staffs. Once the new wing is opened and endowed, the present distressing necessity of sending children home after an anæsthetic, and nursing those who remain in the adult ward or even in the corridors, will be ended. Who that welcomes a restful night in health will not wish to secure it for the patients, young and old, in Harrow Cottage Hospital ?

### NO ROOM IN THE INN.

The Royal Northern Hospital is reported to have been offered a gift of £3,000 if a sum of £5,000 be raised independently by the end of the year. It has, we believe, sixty beds closed, which means that the want of public support has deprived perhaps 1,000 patients of treatment since January. We may probably assume that each closed bed turns away sixteen patients in twelve months. This reflection is so shocking that it should be enough, if properly explained, to raise the relatively small sum asked for in the month that still remains. The work that the voluntary hospitals do is admitted to be the most convincing argument in their favour. Therefore the amount of work that the apathy of the public prevents them from doing is the most convincing plea that can be made. A bed closed for one year for want of funds means the victimisation of sixteen sick people. "How many more do you want to victimise ?" "Are you sure Whom you may not be turning away ?" These are the questions that the Royal Northern Hospital should ask of Mr. Scrooge this Christmas time. The £3,000 will be used, we understand, to reopen twelve beds for maternity cases. If it be not forthcoming before December 25, this hospital will resemble the Inn in the Gospel story because, from it also expectant mothers will, by consent of the public, have been turned away. In a hospital many happy returns are those of the sick to health, and it is the public, and not the hospital, that can withhold them.

### A HASTINGS ENQUIRY.

The Committee of Investigation which was appointed at the annual meeting of the Governors of the Royal East Sussex Hospital, Hastings, has presented its report. It states that, after the first year of experience in the new and larger building, the possibility of economies was realised. An Economy Committee replaced the Contracts Committee, and, during September, was able to save one-third a head on the cost of provisions. To maintain or increase this economy a house steward has been appointed. Mr. R. M. Key fills this post. It is also stated that the settlement of accounts can now be made monthly or quarterly, presumably through an arrangement with the bank whereby the hospital's credit is more promptly convertible into money than before. The kitchen and the warming have been examined by the investigators, who recommend certain suggestions to the House Committee. The rates paid by the hospital are reported to be higher than those of any voluntary hospital in the district. We fear that until the rating system of the country be remodelled, and it will prove a thorny question to tackle, uniformity of treatment for hospitals will remain a pious hope. It is gratifying to learn that the suggestion made at the Governors' meeting that the resident staff were served with "luxurious meals" is unfounded. They have "practically the same dietary as ordinary patients," nor could most hospital kitchens, we fear, ever hope to provide meals in the least likely to satisfy Brillat-Savarin or Lucullus. The typical hospital resident is a gentleman generally in a hurry, with a taste in gastronomy over which the great cooks would have wept.

### THE FUTURE OF ST. LUKE'S, BRADFORD.

The lease of this much-discussed municipal hospital by the Bradford Corporation from the Bradford Guardians has been renewed for five years, at the end of which period the Corporation will no longer be entitled to renew it. It is remarked, however, that, should the draft Rating and Valuation Bill be passed within the next five years, it may grant control of the hospital to the Corporation. But, hypothetical considerations apart, the Guardians are not expected to wish to prevent St. Luke's from continuing. It has been from other quarters that such criticisms as have been made have come.

### A SANATORIUM CITY?

The sanatorium remains, in a sense, the Cinderella of institutions. Wherever one is proposed to be built, objectors arise terribly as an army with banners ; and when one has been built the proposal to establish another is felt to add insult to injury. Yet, if two are more than enough in any one area, clearly a fresh area must be found for each addition ; and if the medical profession quietly insists that the prevention of tuberculosis means the abolition of slums, destitution and overcrowding, the clamour is increased by everyone who can shout at all. Only too often objectors to sanatoria suffer from a disguised fear that their property will depreciate. Therefore, when candid solid arguments are advanced, they should be considered. Three gentlemen in the neighbourhood of Milford, Surrey, object to the plan to build a second sanatorium there on the ground that Dr. Huxley has publicly explained that "the experiment of concentrating sanatoria in one district" has been tried in Switzerland and discarded "because the children in the neighbourhood died in great numbers." The reference is elliptic, but presumably they died of tuberculosis—not of measles. If so, the facts can hardly be too widely known, and the report on which the experiment was discarded deserves careful study. We have never advocated "a sanatorium city" ; but so much exaggeration has been heard when each new sanatorium has been proposed that the story of this Swiss experiment is well worth reviving.

